# Supplementary material for: Does Shiftwork Impact Cognitive Performance? Findings from the Canadian Longitudinal Study on Aging (CLSA)
Source: Int J Environ Res Public Health. 2022 Aug 16;19(16):10124. doi: 10.3390/ijerph191610124 (PMC9408351; doi:10.3390/ijerph191610124)
Supplement: Supplementary file 1 [file ijerph-19-10124-s001.zip › ijerph-1840534-supplementary.pdf]

Supplementary Table S1. Weighted sample characteristics, stratified by retirement status and sex (N=22,485)

Characteristic	All (N= 22,485)	Males		Females	
		Completely Retired (n=5,484)	Not/Partly Retired (n=6,886)	Completely Retired (n=5,149)	Not/Partly Retired (n=4,966)
	%	%	%	%	%
**Shift Work Status**					
No shift work	82.8	79.9	82.2	85.1	83.8
Shift work	17.2	20.1	17.8	14.5	16.2
Missing	0.0	0.0	0.0	0.4	-
**Age (years)**					
Mean (SD)	60.0 (10.2)	69.0 (8.2)	54.5 (7.2)	68.4 (8.3)	54.0 (6.6)
45 -54 years	37.6	2.8	59.5	4.9	60.1
55 -64 years	31.4	30.1	31.3	30.6	33.3
65 - 74 years	19.2	38.5	7.5	38.0	5.5
75 – 85 years	11.8	28.5	1.7	26.5	1.1
**Education**					
Less than secondary	17.3	27.7	11.4	28.1	8.8
Secondary	11.5	11.7	10.1	14.3	11.0
Some post-secondary	8.9	9.2	8.7	9.8	8.3
Post-secondary	62.2	51.1	69.8	47.8	71.8
Missing	0.0	0.3	0.0	0.0	0.0

Acronyms: SD: Standard deviation.
Note: Multimorbidity is defined as $\geq$ 2 of the following conditions: anxiety or depression, cancer, cardiovascular disease, asthma, COPD, diabetes, arthritis, stroke.

Supplementary Table S1 (continued)

Characteristic	All (N= 22,485)	Males		Females	
		Completely Retired (n=5,484)	Not/Partly Retired (n=6,886)	Completely Retired (n=5,149)	Not/Partly Retired (n=4,966)
	%	%	%	%	%
**Household Income**					
Less than $20,000	4.3	6.8	1.8	9.1	1.9
$20,000 - $49,999	20.4	30.2	10.5	36.1	14.1
$50,000 - $99,999	32.3	38.5	29.6	30.3	32.9
$100,000 - $149,999	20.2	12.3	27.6	9.1	25.2
Greater than $150,000	17.1	6.3	27.2	4.8	21.3
Missing	5.6	5.9	3.4	10.6	4.6
**Marital Status**					
Single, never married, or never lived with a partner	7.8	6.4	7.7	7.3	9.4
Married or common law	75.2	78.5	84.7	60.5	71.3
Widowed, divorced or separated	17.0	15.1	7.6	32.2	19.1
Missing	0.0	-	0.0	0.0	0.2
**Migrant Status**					
Non-Immigrant	81.3	80.3	80.8	81.9	82.3
Immigrant	11.9	14.4	11.2	12.3	10.5
Missing	6.8	5.3	8.0	5.8	7.2

Acronyms: SD: Standard deviation.
Note: Multimorbidity is defined as $\geq$ 2 of the following conditions: anxiety or depression, cancer, cardiovascular disease, asthma, COPD, diabetes, arthritis, stroke.

Supplementary Table S1 (continued)

Characteristic	All (N= 22,485)	Males Completely Retired (n=5,484)	Males Not/Partly Retired (n=6,886)	Females Completely Retired (n=5,149)	Females Not/Partly Retired (n=4,966)
	%	%	%	%	%
**Place of Residence**					
Rural	5.1	4.8	4.7	4.7	6.2
Urban	90.4	90.6	90.5	90.9	89.9
Suburban	4.5	4.6	4.8	4.4	3.9
**Socially Isolated**					
No	79.2	56.9	91.0	60.9	94.6
Yes	20.7	42.8	9.0	38.8	5.4
Missing	0.1	0.3	0.0	0.3	0.0
**Smoking Status**					
Never smoker	30.0	21.6	30.7	32.1	33.6
Former smoker	58.9	68.5	58.7	57.3	53.1
Occasional or daily smoker	10.7	9.3	10.4	9.6	12.9
Missing	0.5	0.6	0.3	1.0	0.4
**Past Week Physical Activity (PASE scale: 0 - 693)**					
Mean (Standard Deviation)	152.0 (78.7)	125.2 (59.3)	182.5 (83.3)	110.1 (54.2)	163.1 (80.1)
Missing (n)	1,138	314	303	281	240

Acronyms: SD: Standard deviation.
Note: Multimorbidity is defined as $\geq$ 2 of the following conditions: anxiety or depression, cancer, cardiovascular disease, asthma, COPD, diabetes, arthritis, stroke.

Supplementary Table S1 (continued)

Characteristic	All (N= 22,485)	Males: Completely Retired (n=5,484)	Males: Not/Partly Retired (n=6,886)	Females: Completely Retired (n=5,149)	Females: Not/Partly Retired (n=4,966)
	%	%	%	%	%
**Alcohol Consumption**					
Never drinker	2.6	2.1	1.6	4.6	2.6
Former drinker	13.1	14.0	10.6	18.2	11.9
Infrequent drinker	11.8	9.3	8.7	17.0	14.0
Occasional drinker	25.7	22.4	26.3	23.9	28.8
Regular drinker	43.5	48.9	48.3	34.7	39.8
Binge drinker	3.3	3.3	4.5	1.6	2.8
Missing	0.0	0.0	0.0	0.0	0.1
**Fruit and Vegetable Intake (Servings/ day)**					
Seven or more	10.2	7.5	7.6	12.1	14.4
Six	9.5	6.6	7.3	12.5	12.1
Five	16.1	11.9	13.7	18.2	20.8
Four	16.2	13.6	14.9	18.4	18.4
Three	16.5	18.2	18.6	14.1	14.2
Two	14.8	18.5	18.4	11.2	10.1
Less than two	10.3	16.3	13.8	6.3	4.2
Missing	6.3	7.4	5.6	7.2	5.8

Acronyms: SD: Standard deviation.
Note: Multimorbidity is defined as $\geq$ 2 of the following conditions: anxiety or depression, cancer, cardiovascular disease, asthma, COPD, diabetes, arthritis, stroke.

Supplementary Table S1 (continued)

Characteristic	All (N= 22,485)	Males: Completely Retired (n=5,484)	Males: Not/Partly Retired (n=6,886)	Females: Completely Retired (n=5,149)	Females: Not/Partly Retired (n=4,966)
	%	%	%	%	%
**BMI (kg/m^2)**					
Underweight or normal weight	28.0	22.4	22.4	30.8	37.6
Overweight	39.6	43.4	45.4	36.8	30.8
Obese	31.1	32.4	30.9	31.4	30.3
Missing	1.3	1.8	1.3	1.0	1.3
**Self-rated Health**					
Excellent or Good Self-Rated Health	91.1	86.7	92.2	89.6	94.1
Fair or Poor Self-Rated Health	8.9	13.3	7.7	10.4	5.9
Missing	0.0	0.0	0.1	0.0	0.0
**Multimorbidity**					
0 -1 Chronic Disease	72.1	63.5	83.3	57.8	74.6
≥ 2 Chronic Diseases	27.9	36.5	16.7	42.2	25.4
**Psychological Distress**					
Low Distress	81.9	82.8	85.0	77.6	80.4
High Distress	12.1	10.0	10.3	15.2	13.7
Missing	6.0	7.2	4.7	7.2	5.9
**Sleep Quality**					
Good Sleep Quality	74.1	79.1	75.4	72.7	69.6
Poor Sleep Quality	25.8	20.8	24.5	27.2	30.4
Missing	0.0	0.2	0.0	0.1	0.0

Acronyms: SD: Standard deviation.
Note: Multimorbidity is defined as ≥ 2 of the following conditions: anxiety or depression, cancer, cardiovascular disease, asthma, COPD, diabetes, arthritis, stroke.

Supplementary Table S2. Weighted sample characteristics of shiftworkers by sex (N=3,632)

Characteristic	All (N= 3,632)	Males (n=2,131)	Females (n=1,501)
	%	%	%
**Age (years)**			
Mean (SD)	60.1 (10.0)	60.4 (10.0)	59.7 (10,2)
45 -54 years	35.2	34.8	35.8
55 -64 years	34.2	33.3	35.6
65 - 74 years	19.8	20.7	18.4
75 – 85 years	10.8	11.2	10.2
**Education**			
Less than secondary	24.0	24.7	23.2
Secondary	11.1	13.0	8.5
Some post-secondary	9.7	11.5	7.1
Post-secondary	55.1	50.9	61.1
Missing	0.0	0.0	0.0
**Household Income**			
Less than $20,000	6.8	4.7	9.9
$20,000 - $49,999	24.1	20.6	29.1
$50,000 - $99,999	32.4	33.9	30.3
$100,000 - $149,999	17.6	19.5	14.9
Greater than $150,000	14.2	17.0	10.2
Missing	4.8	4.3	5.5

Acronyms: SD: Standard deviation.
Note: Multimorbidity is defined as $\geq$ 2 of the following conditions: anxiety or depression, cancer, cardiovascular disease, asthma, COPD, diabetes, arthritis, stroke.

Supplementary Table S2 (continued)

Characteristic	All (N= 3,632)	Males (n=2,131)	Females (n=1,501)
	%	%	%
**Marital Status**			
Single, never married, or never lived with a partner	9.1	7.8	11.0
Married or common law	73.9	81.3	63.4
Widowed, divorced or separated	16.8	10.9	25.3
Missing	0.0	-	0.2
**Migrant Status**			
Non-Immigrant	79.2	79.9	78.2
Immigrant	11.9	12.0	11.8
Missing	8.9	8.1	10.0
**Place of Residence**			
Rural	6.6	7.0	6.0
Urban	88.9	87.9	90.3
Suburban	4.4	5.0	3.6
**Socially Isolated**			
No	79.4	78.6	80.5
Yes	20.3	21.0	19.3
Missing	0.3	0.4	0.2

Acronyms: SD: Standard deviation.
Note: Multimorbidity is defined as ≥ 2 of the following conditions: anxiety or depression, cancer, cardiovascular disease, asthma, COPD, diabetes, arthritis, stroke.

Supplementary Table S2 (continued)

Characteristic	All (N= 3,632)	Males (n=2,131)	Females (n=1,501)
	%	%	%
**Smoking Status**			
Never smoker	31.3	27.5	36.8
Former smoker	56.8	60.9	50.8
Occasional or daily smoker	11.4	11.3	11.7
Missing	0.4	0.3	0.7
**Past Week Physical Activity (PASE scale: 0 - 693)**			
Mean (Standard Deviation)	163.6 (85.6)	168.4 (85.9)	156.5 (84.7)
Missing (n)	218	121	97
**Alcohol Consumption**			
Never drinker	2.8	2.4	3.4
Former drinker	17.2	15.7	19.2
Infrequent drinker	12.7	9.8	16.7
Occasional drinker	25.8	25.4	26.3
Regular drinker	37.9	41.8	32.4
Binge drinker	3.6	4.7	1.9
Missing	0.0	0.0	0.1

Acronyms: SD: Standard deviation.
Note: Multimorbidity is defined as ≥ 2 of the following conditions: anxiety or depression, cancer, cardiovascular disease, asthma, COPD, diabetes, arthritis, stroke.

Supplementary Table S2 (continued)

Characteristic	All (N= 3,632)	Males (n=2,131)	Females (n=1,501)
	%	%	%
**Fruit and Vegetable Intake (Servings/ day)**			
Seven or more	10.1	8.0	13.2
Six	9.1	9.2	9.1
Five	14.9	11.0	20.6
Four	15.8	13.4	19.1
Three	14.0	15.6	11.8
Two	16.9	20.9	11.3
Less than two	11.6	14.9	6.7
Missing	7.5	7.1	8.2
**BMI (kg/m^2)**			
Underweight or normal weight	24.3	18.4	32.7
Overweight	39.7	44.9	32.2
Obese	34.6	34.9	34.2
Missing	1.4	1.8	0.8
**Self-rated Health**			
Excellent or Good Self-Rated Health	88.8	87.3	91.0
Fair or Poor Self-Rated Health	11.1	12.6	9.0
Missing	0.0	0.0	0.0

Acronyms: SD: Standard deviation.
Note: Multimorbidity is defined as ≥ 2 of the following conditions: anxiety or depression, cancer, cardiovascular disease, asthma, COPD, diabetes, arthritis, stroke.

Supplementary Table S2 (continued)

Characteristic	All (N= 3,632)	Males (n=2,131)	Females (n=1,501)
	%	%	%
**Multimorbidity**			
0 -1 Chronic Disease	69.6	73.0	64.6
≥ 2 Chronic Diseases	30.4	27.0	35.4
**Psychological Distress**			
Low Distress	81.4	82.9	79.1
High Distress	11.9	10.6	13.9
Missing	6.7	6.5	7.0
**Sleep Quality**			
Good Sleep Quality	73.0	74.3	71.1
Poor Sleep Quality	27.0	25.6	28.9
Missing	0.0	0.0	0.0

Acronyms: SD: Standard deviation.
Note: Multimorbidity is defined as ≥ 2 of the following conditions: anxiety or depression, cancer, cardiovascular disease, asthma, COPD, diabetes, arthritis, stroke.

Supplementary Table S3. Weighted descriptive analysis of cognition scores among shiftworkers by sex (N=3,312).

Cognition scores	All (N= 3,312)	Males (n=1,932)	Females (n=1,380)
	**n**	**n**	**n**
**Immediate Recall (0 – 14 points)**			
Mean (SD)	5.6 (1.8)	5.3 (1.71)	6.1 (1.9)
**Delayed Recall (0 – 14 points)**			
Mean (SD)	3.9 (2.0)	3.5 (1.9)	4.4 (2.1)
**MAT Score (0 – 51 points)**			
Mean (SD)	25.7 (9.0)	26.4 (9.0)	24.7 (8.9)
**Interference Condition (1-132 seconds)**			
Mean (SD)	16.7 (5.0)	16.8 (4.9)	16.5 (5.0)

Acronyms: SD: Standard deviation.

Supplementary Table S4. Interaction effect between psychological distress and shift work

	ß	95% CI
**Immediate Recall Trial**		
Shift Work x High Distress	0.22	-0.05, 0.48
**Delayed Recall Trial**		
Shift Work x High Distress	0.09	-0.19, 0.37
**MAT**		
Shift Work x High Distress	-0.36	-1.57, 0.85
**Interference Condition**		
Shift Work x High Distress	0.60	-0.08, 1.28

Abbreviations: CI: confidence interval;
Significant results are bolded.
